# Application of optical coherence tomography angiography to assess systemic severity in patients with hereditary transthyretin amyloidosis

**DOI:** 10.1371/journal.pone.0275180

**Published:** 2022-09-26

**Authors:** Shinji Kakihara, Takao Hirano, Junya Kitahara, Yorishige Matsuda, Akira Imai, Teruyoshi Miyahara, Toshinori Murata

**Affiliations:** Department of Ophthalmology, Shinshu University School of Medicine, Matsumoto, Nagano, Japan; Massachusetts Eye & Ear Infirmary, Harvard Medical School, UNITED STATES

## Abstract

Hereditary transthyretin amyloidosis is an autosomal dominant form of amyloidosis caused by an abnormality in transthyretin, with various ocular manifestations. Among these, ocular amyloid angiopathy has attracted attention because of its direct link to visual impairment and its correlation with systemic severity. We hypothesized that optical coherence tomography angiographic parameters would be useful biomarkers of amyloidosis systemic severity and investigated their correlation with the systemic severity score. The primary outcome was the correlation between the systemic severity score and choriocapillaris flow deficit percentage. Secondary outcomes were the correlations between the systemic severity score and retinal optical coherence tomography angiographic parameters, including foveal avascular zone size and circularity and superficial/deep/total retinal perfusion and vessel densities. The choroidal and retinal vasculature was quantified in 36 eyes from 36 patients (age, 51.8±12.1 years; disease duration, 13.4±6.2 years). Ten eyes had a history of vitrectomy for vitreous opacity. Choriocapillaris flow deficit percentage was not significantly correlated with the systemic severity score (Spearman’s rank correlation: r = 2.96×10^−2^, p = 0.863). Similarly, foveal avascular zone size and circularity, and superficial/deep/total retinal perfusion and vessel densities were not significantly correlated with the systemic severity score. These results may indicate that optical coherence tomography angiographic parameters are not sufficient to predict amyloidosis severity.

## Introduction

Amyloidosis comprises a group of diseases in which amyloid fibrils are deposited in various organs. There are several types of systemic amyloidosis, including inflammatory, light chain-related, dialysis-related, and hereditary amyloidosis [1]. Hereditary amyloidosis, or familial amyloid polyneuropathy, comprises a group of rare and fatal diseases frequently associated with disorders of the nervous system and heart, usually due to mutations in the gene encoding transthyretin (TTR) [1]. TTR-related familial amyloid polyneuropathy, normally referred to as hereditary amyloidogenic TTR (hATTR) amyloidosis, is an autosomal dominant disorder with a clinical onset usually before the age of 50 years and an overall survival time of less than 10 years if untreated [2]. Among the TTR variants, TTR Val30Met is the most well-studied and is highly prevalent in Portugal, Sweden, Japan, and other countries [2]. Ocular symptoms such as irregular pupil, dry eye, vitreous opacity, glaucoma, and amyloid angiopathy are often observed in hATTR amyloidosis [3–15]. Effective systemic treatments, including liver transplantation and disease-modifying drugs (tafamidis and patisiran), slow disease progression and increase the survival rate among patients with hATTR amyloidosis; however, the frequencies of ocular symptoms are increased because TTR is produced in the liver and eyes [16–19].

Among the ocular manifestations, ocular amyloid angiopathy is an important presentation because it can cause serious complications, such as neovascular glaucoma. In 2005, Kawaji et al. [20] reported on ocular amyloid angiopathy in patients with hATTR Tyr114Cys amyloidosis, a severe and progressive form of amyloid angiopathy causing visual impairment, and brought ocular amyloid angiopathy to the attention of clinicians. Recently, several studies reported on choroidal amyloid angiopathy detected by indocyanine green angiography in patients with systemic and hATTR amyloidosis as a useful biomarker of systemic amyloidosis severity [21–23]. Recently, we reported for the first time that retinal amyloid angiopathy, which can be detected by fluorescein angiography, is also correlated with the systemic severity of amyloidosis [21].

In recent years, optical coherence tomography angiography (OCTA), which enables the quantification of fundus vessel perfusion non-invasively, has become a useful tool for understanding and diagnosing the pathogenesis of various ocular diseases and as a biomarker for non-ocular diseases such as Alzheimer’s disease [24, 25]. In patients with hATTR amyloidosis, OCTA has been reported as useful in detecting early changes in the retinal vessels and evaluating amyloid oculopathy [26]. Based on these previous reports, we hypothesized that OCTA parameters, especially a choroidal parameter, would be useful biomarkers of systemic severity in patients with amyloidosis.

Therefore, in the current study, we retrospectively investigated the relationships between OCTA parameters and systemic severity in patients with hATTR amyloidosis.

## Results

The demographic and clinical characteristics of the study participants are summarized in Table 1. The mean age (± the standard deviation) was 51.8±12.1 years. The mean duration from disease onset was 13.4±6.2 years. Ten eyes underwent previous treatment with pars plana vitrectomy. Thirty patients had the TTR Val30Met variant; the remaining six patients had other variants (Ser50Arg, Asp38Ala, Tyr114His, Ser50Ile, Arg54Thr, and Phe53Val). Systemic treatments included liver transplantation in 17 patients, tafamidis administration in 10 patients (seven eyes), and patisiran administration in seven patients. The mean systemic severity score was 6.97±2.85.

**Table 1:** Demographic and clinical characteristics of the 36 patients.

Characteristic		Value
Age (years)		51.8±12.1
Duration of disease (years)		13.4±6.2
Eyes with a history of PPV		10 eyes
Systemic severity score		6.97±2.85
TTR variant	Val30Met	30 patients
	Others	6 patients
Systemic treatment	Liver transplantation	17 patients
	Administration of tafamidis	10 patients
	Administration of patisiran	7 patients
	Others	2 patients
Sex	Male/Female	22/14

Data are displayed as mean ± standard deviation. PPV, pars plana vitrectomy

Ocular measurements are summarized in Table 2. The mean signal strength was 8.69±0.86 (range, 7–10). The mean logarithm of the minimum angle of resolution, best-corrected visual acuity was -0.127±0.09, and the mean axial length (AL) was 24.33±1.07 mm.

**Table 2:** Ocular measurements of the 36 patients.

Ocular measurement	Value
LogMAR BCVA	-0.127±0.09
Axial length (mm)	24.33±1.07
Signal strength	8.69±0.86
Choriocapillaris flow deficits (%)	28.6±9.2
FAZ size (mm^2^)	0.445±0.266
FAZ circularity	0.689±0.114
Superficial perfusion density	0.330±0.054
Deep perfusion density	0.197±0.084
Total retinal perfusion density	0.360±0.055
Superficial vessel density (mm^-1^)	13.31±2.32
Deep vessel density (mm^-1^)	8.42±3.41
Total retinal vessel density (mm^-1^)	14.69±2.37

Data are displayed as mean ± standard deviation. LogMAR BCVA, logarithm of the minimum angle of resolution, best-corrected visual acuity; FAZ, foveal avascular zone

**Fig 1:**
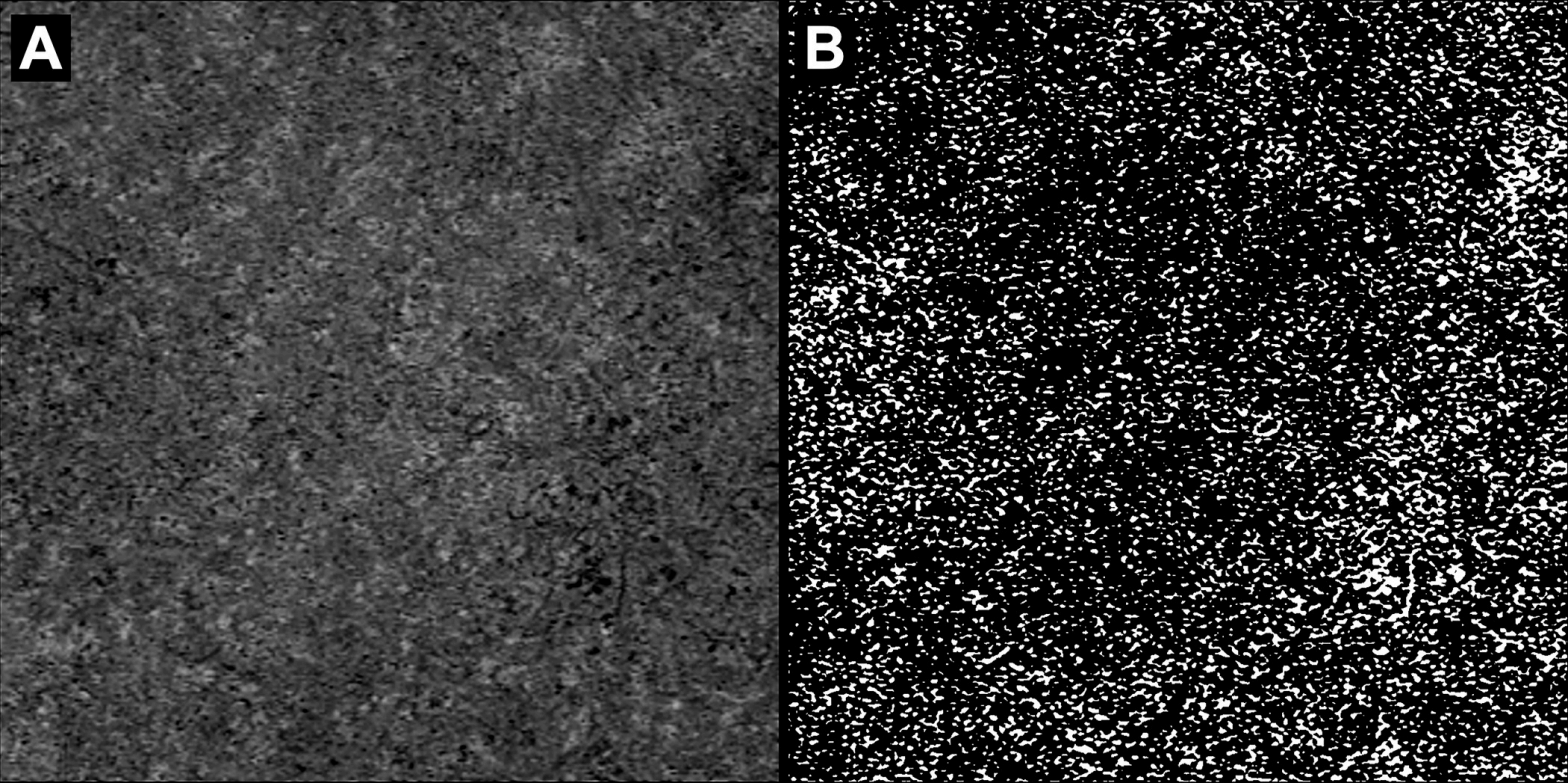
*En face* swept-source OCTA choriocapillaris images before and after automatic local thresholding are shown. (A) A choriocapillaris image obtained by a choriocapillaris slab with 20-μm thickness at 29–49 μm beneath the retinal pigment epithelium fit layer, with projection artifacts removal, is shown. (B) A choriocapillaris image thresholded with the Phansalkar method, using a radius of 6 pixels, is shown. OCTA, optical coherence tomography angiography.

**Fig 2:**
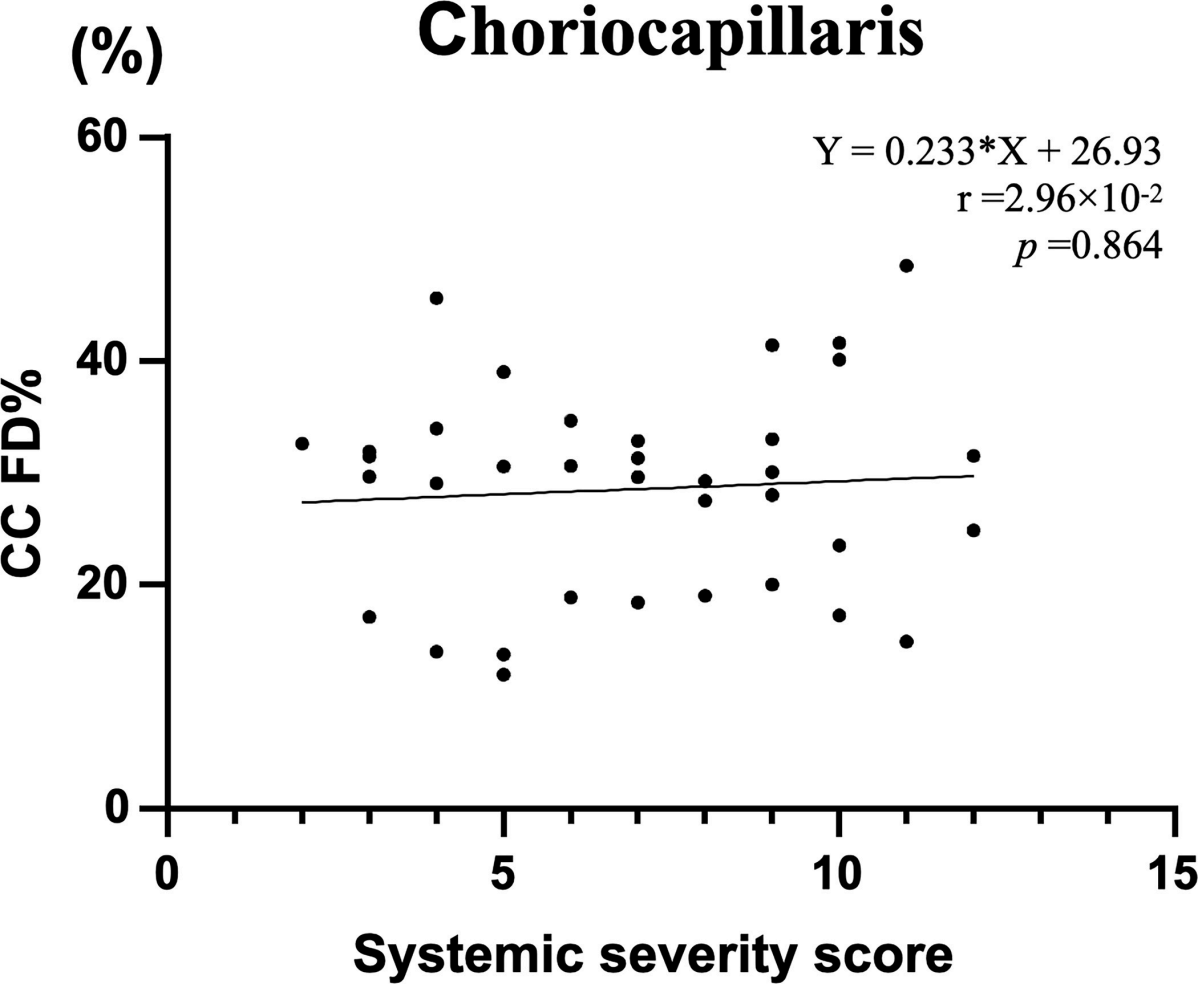
Spearman’s rank correlation analysis showed no significant correlation between choriocapillaris flow deficit percentage (CC FD%) and the systemic severity score.

Fig 1 shows an example of the choriocapillaris analysis, and Fig 2 illustrates the primary outcome of this study. The mean choriocapillaris flow deficit percentage (CC FD%) was 28.6±9.2. CC FD% was not significantly correlated with the systemic severity score (Spearman’s rank correlation: r = 2.96×10^−2^, p = 0.863). No statistical correlations were observed in the multivariate analysis (Table 3). We confirmed that similar results were obtained using global mean binarization thresholding (S1 File).

**Table 3:** Univariate and multivariate analyses for the relationship between the systemic severity score and parameters.

Parameters	Univariate Analysis		Multivariate Analysis		
	R (95% CI)	P value	β (95% CI)	Test Statistic	P Value
CC FD%	0.030 (-0.31 to 0.36)	0.864	0.029 (-0.086 to 0.145)	0.517	0.609
Age	-0.038(-0.37 to 0.30)	0.826	-0.014 (-0.103 to 0.074)	0.328	0.745
Signal strength	-0.086(-0.41 to 0.26)	0.618	-0.30(-1.492 to 0.893)	0.513	0.612

CC FD%, choriocapillaris flow deficits percentage; CI, confidence interval

**Fig 3:**
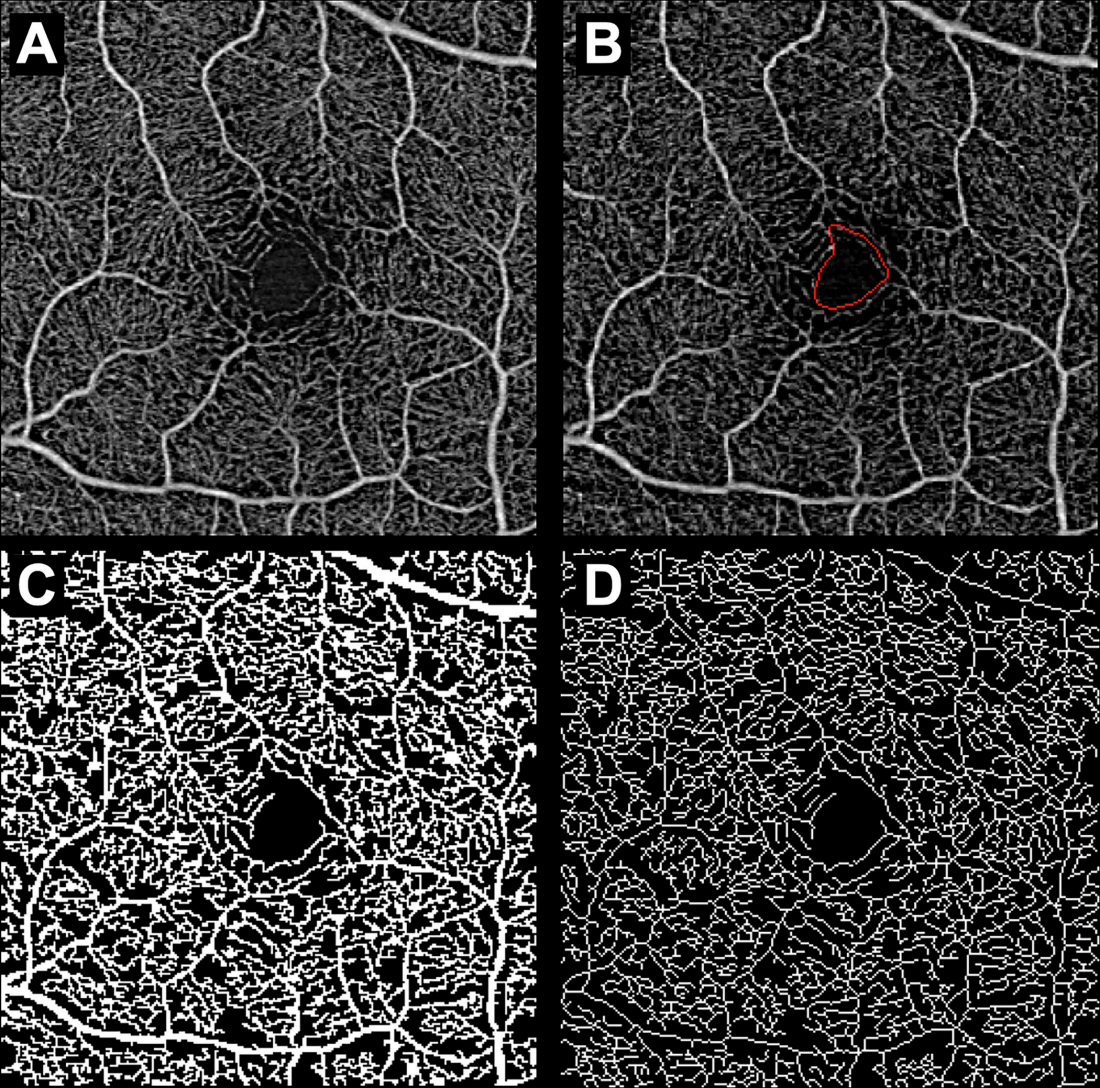
*En face* swept-source OCTA retinal images analyzed by Advanced Retina Imaging Network algorithms are shown. (A) The total retinal slab is shown. (B) Automated identification of the foveal avascular zone in the superficial retina is shown. (C) Automated identification of the superficial *en face* perfusion trace is shown. (D) Automated identification of the superficial *en face* vessel trace is shown. OCTA, optical coherence tomography angiography.

**Fig 4:**
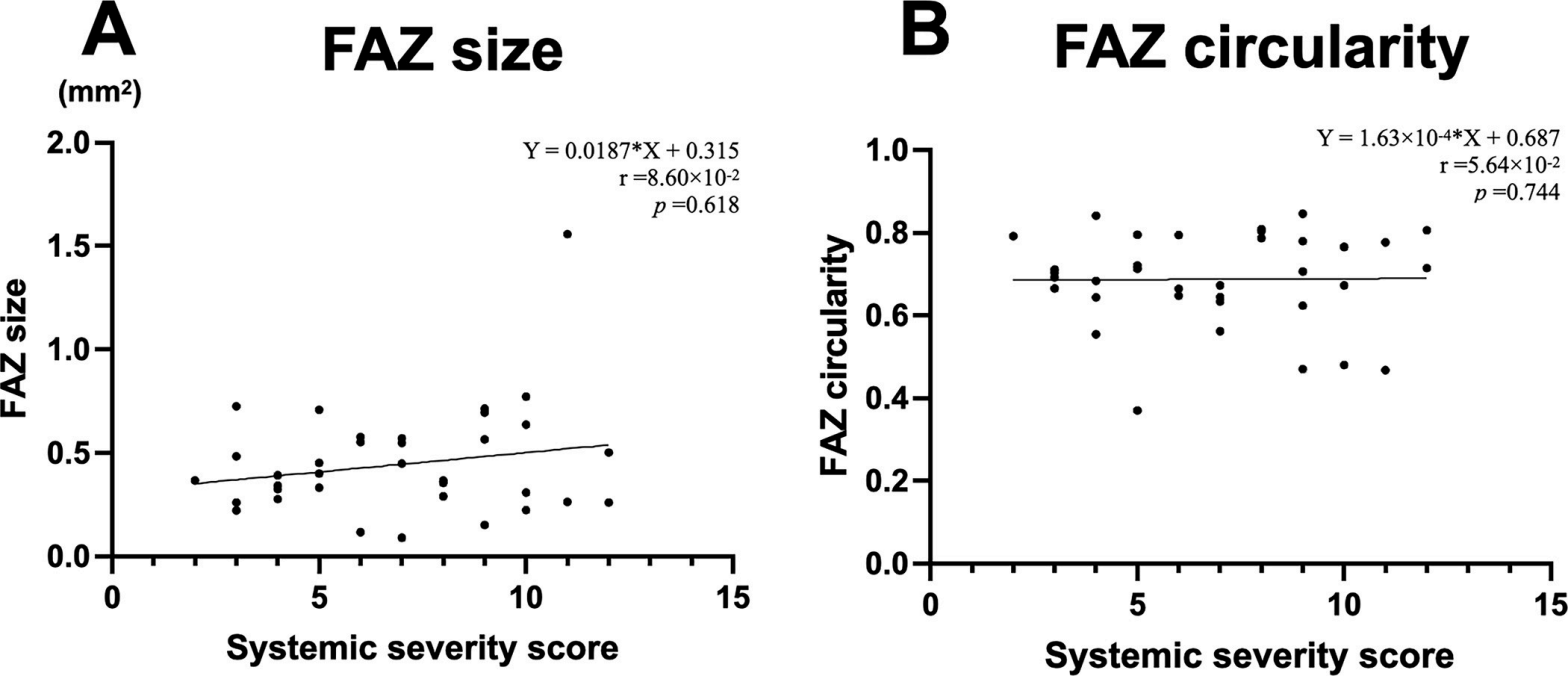
Spearman’s rank correlation analysis showed no significant correlations between the foveal avascular zone (FAZ) parameters ((A) FAZ size; (B) FAZ circularity) and the systemic severity score.

**Fig 5:**
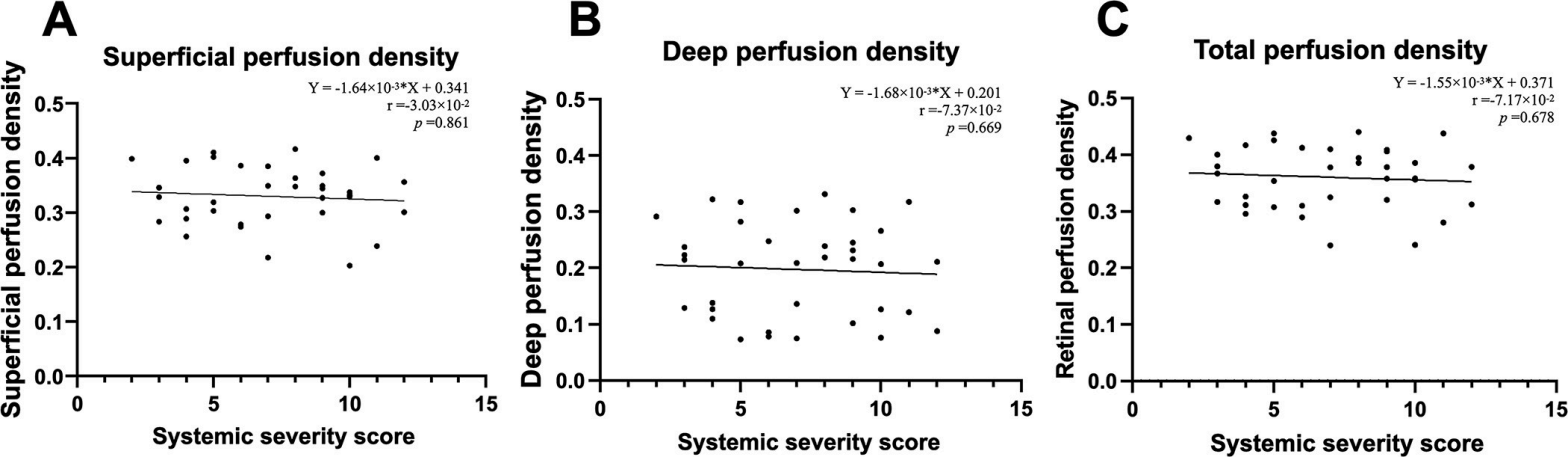
Spearman’s rank correlation analysis showed no significant correlations between perfusion density in various slabs ((A) superficial slab; (B) deep slab; (C) total retinal slab) and the systemic severity score.

**Fig 6:**
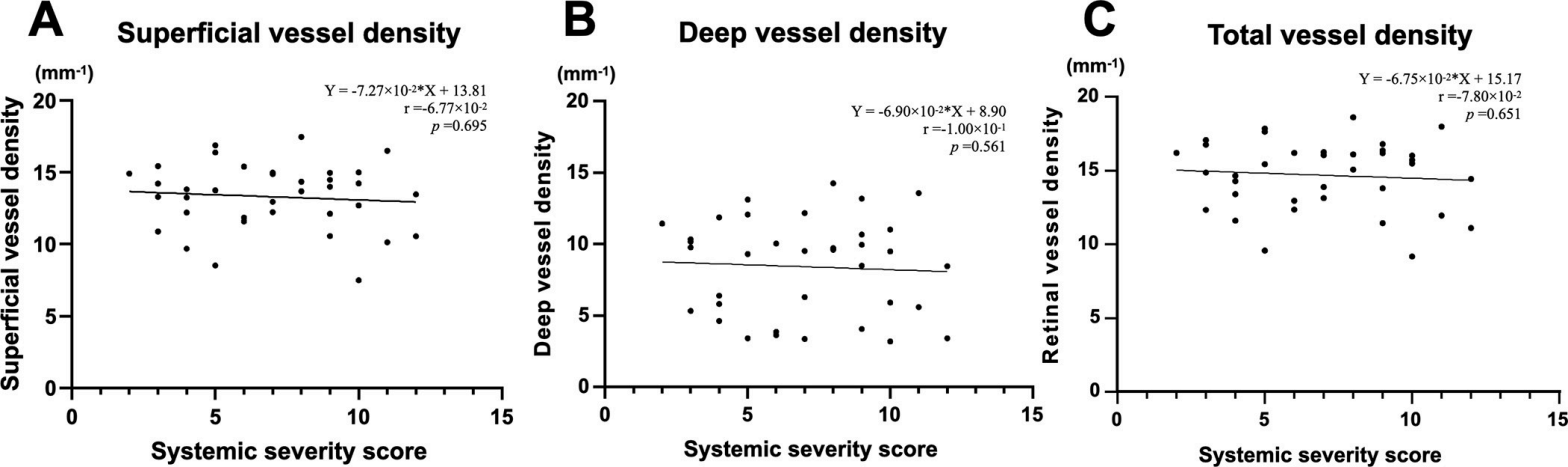
Spearman’s rank correlation analysis showed no significant correlations between the vessel density in various slabs ((A) superficial slab; (B) deep slab; (C) total retinal slab) and the systemic severity score.

Fig 3 shows an example of the automated retinal layer analysis, and Figs 4–6 summarize the secondary outcomes of this study. Fig 4 depicts the correlation (Spearman’s rank) between the foveal avascular zone (FAZ) parameters and systemic severity. The systemic severity score was not significantly correlated with FAZ size (Fig 4A, r = 8.60×10^−2^, p = 0.617) or FAZ circularity (Fig 4B, r = 5.64×10^−2^, p = 0.744). Fig 5 depicts the correlations (Spearman’s rank) between perfusion density in various slabs and systemic severity. The systemic severity score was not significantly correlated with superficial/deep/total retinal perfusion density (Fig 5A, r = -3.02×10^−2^, p = 0.861; Fig 5B, r = -7.37×10^−2^, p = 0.669; and Fig 5C, r = -7.17×10^−2^, p = 0.678, respectively). Fig 6 depicts the correlation (Spearman’s rank) between vessel density in various slabs and systemic severity. The systemic severity score was also not significantly correlated with superficial/deep/total retinal vessel density (Fig 6A, r = -6.77×10^−2^, p = 0.695; Fig 6B, r = -1.00×10^−1^, p = 0.561; and Fig 6C, r = -7.80×10^−2^, p = 0.651, respectively).

## Discussion

We predicted that parameters quantified by OCTA, especially CC FD%, would be useful biomarkers for predicting the systemic severity of amyloidosis; however, we could not confirm any correlation between the OCTA parameters and the systemic severity score.

There may be several reasons for the lack of correlation. First, the participants underwent different systemic treatments, which may have influenced the results. Second, retinal amyloid angiopathy tends to start primarily in the peripheral retina on fluorescein angiography [21]; thus, macular vessel changes may be less likely to occur at first. Third, there may be data variability that was not considered in the present study. For example, this study did not compare the left and right eyes of the same patient and did not analyze measurements over time in the same eyes. Most importantly, a previous pathological study demonstrated that amyloid deposits are predominantly found on the outer wall of retinal/choroidal vessels; accordingly, it may be that the inside of the vessels is not affected by amyloid deposits until much later in the course of amyloid oculopathy [20, 28]. In amyloidosis other than hATTR amyloidosis, amyloid fibrils are thought to be deposited on the vessels themselves rather than in the lumen [27]. The results of the above-mentioned pathological study also imply that amyloid deposition is predominantly detected in large vessels, not in capillaries [20, 28]. Since OCTA reflects the red blood cells flowing in vessels, OCTA parameters may not be affected until amyloidosis is severe, or in its late or end stages [29]. A recent report showing no significant differences in macular vascular density or FAZ parameters between eyes with familial Mediterranean fever, an inherited autoinflammatory disease with amyloidosis and vasculitis, and age- and gender-matched normal eyes support the results of this study [30].

Mano et al. [22] graded systemic amyloidosis by focusing on the signal strength of the choroidal vessels on enhanced depth imaging optical coherence tomography. Considering the above-mentioned pathological study [20], it is reasonable to assume that choroidal vascular intensity measured by B-scan optical coherence tomography, especially for Sattler’s layer, may be a useful biomarker of amyloidosis, unlike OCTA parameters.

The present study had several limitations. It was a retrospective case series study, and most patients were Japanese. OCTA images acquired with a recent update to a 200-kHz A-scan rate were not utilized, and only images with a 3×3-mm scan size were analyzed. It cannot be ruled out that wider OCTA scans or OCTA scans without centering on macula may have been better for evaluation [31, 32]. We did not apply RPE attenuation correction, correction for signal strength, or any other normalization methods, which have been reported as useful, and utilized a single threshold algorithm [33–37]. Longitudinal studies are essential to accurately determine the usefulness of OCTA in this disease. Since this study is a cross-sectional study, it is undeniable that we are potentially missing microvascular changes in this study. The equipment used in this study is not the latest OCTA equipment. Eyes with vitreous opacity were excluded; therefore, patients with certain stages of ocular amyloidosis were excluded. The influence of needle-shaped deposits on the retinal surface may not have been completely excluded in some cases [9]. Only patients with hATTR amyloidosis were studied; other types of amyloidosis were not studied.

In summary, contrary to our expectations, OCTA quantitative assessments do not provide useful biomarkers for systemic amyloidosis severity. To the best of our knowledge, this is the first study to evaluate the relationships between OCTA parameters and systemic severity in patients with systemic amyloidosis in an attempt to identify a useful biomarker to assess amyloidosis.

## Methods

This retrospective study reviewed 56 patients with hATTR amyloidosis who were examined with 3×3-mm swept-source OCTA, centered on the fovea, using PLEX Elite 9000 (Carl Zeiss Meditec Inc., Dublin, California, USA) operating at a 100-kHz A-scan rate, between October 2016 and September 2021 as part of an ordinary examination. This instrument employed a full width at a half-maximum axial resolution of approximately 6 μm in tissue and a lateral resolution at a retinal surface of approximately 20 μm [38]. OCTA images with the following features were excluded due to poor quality: signal strength less than 7, motion or blinking artifacts, local signal weakness (due to vitreous opacities), or wrong segmentation of retinal or choroidal slabs. OCTA images acquired with a recent update to a 200-kHz A-scan rate were also excluded. The results of the eye with higher signal strength in the image were utilized; if the signal strength was the same in both eyes, the results of the right eye were utilized. AL was measured with IOL Master (Carl Zeiss Meditec Inc., Dublin, California, USA) or A-mode ultrasound US-500(Nidek Co., Gamagori Aichi, Japan); if both were available, the IOL Master measurement was utilized. Eyes with AL greater than 26.5 mm or less than 22.0 mm were excluded from the analysis. Eyes with ocular diseases unrelated to amyloid oculopathy, such as diabetic retinopathy and age-related macular degeneration, were also excluded. As a result of the inclusion and exclusion criteria, 36 eyes from 36 patients were included in the study. The diagnosis of all patients was confirmed by the Department of Medicine (Neurology and Rheumatology) at Shinshu University School of Medicine, and the patients were referred to the Department of Ophthalmology for ocular amyloidosis evaluation. Data on the TTR variant, age at disease onset, systemic presentations, and systemic treatment were obtained from medical history records.

In order to quantify the OCTA parameters, we first uploaded raw data to the Advanced Retina Imaging Network on the Advanced Retina Imaging Network Hub (https://arinetworkhub.com). The data were then analyzed using Standard Visualization (v3.08) and Macular Density (v0.7.3.3) algorithms, which automatically quantified the superficial, deep, and total retinal slabs [39]. The superficial slab was defined as the slab from the internal limiting membrane to the inner plexiform layer. The deep slab was defined as the slab from the inner plexiform layer to the outer plexiform layer. The total retinal slab was defined as the slab from the internal limiting membrane to the layer 41 μm above Bruch’s membrane. The choriocapillaris OCTA image was obtained using a choriocapillaris slab with 20-μm thickness at 29–49 μm beneath the retinal pigment epithelium fit layer, with projection artifact removal and an image size of 1024×1024 pixels. CC FD% was measured according to the latest guidelines, using Fuji software (National Institutes of Health, Bethesda, Maryland, USA) [40]. The Phansalkar threshold method was applied to binarize the choriocapillaris images, with a radius of 6 pixels, to optimize the image [35]. One to two times the intercapillary distance has been proposed as the optimal window diameter (= 2 × radius + 1 pixel) [40]. Power spectral analysis to calculate the intercapillary distance in averaged CC images showed an average intercapillary distance of 23.17 μm (95% confidence interval: 21.05–25.28 μm) under the fovea for 3×3- mm OCTA images [41]. Thus, a window radius of 4–8 pixels is appropriate; we chose a window radius of 6 pixels to optimize the image parameters.

To quantify CC FD%, the CC slab images were imported into Fuji software. Then

select File>New>script and paste the following:

run("8-bit");

run("Auto Local Threshold", "method = Phansalkar radius = 6 parameter_1 = 0 parameter_2 = 0");

run("Analyze Particles…", "summarize");

followed by select Language>IJ1 Macro and click “run.” We used global mean binarization thresholding to confirm whether other thresholding methods showed a similar trend. The magnification effect due to AL was corrected by using Littmann’s method and Bennett’s formula [42]. The PLEX Elite 9000 assumes an eye with an AL of 22.0 mm as the standard.

The severity of systemic amyloidosis was determined near the time of OCTA acquisition. Systemic severity scores were retrospectively calculated by examining medical charts for heart, liver, renal, gastrointestinal, and neural impairments, as previously described [21, 22]. Systemic severity scores were evaluated for each organ and summed for each case: 0, no impairment; 1, abnormal data but asymptomatic; 2, symptomatic complications; and 3, severe disability (including a history of organ transplantation). One evaluator (J.K.) reviewed the medical charts and calculated the systemic severity score for each patient.

The primary outcome was the correlation between the systemic severity score and CC FD%. Secondary outcomes were the correlations between the systemic severity score and retinal OCTA parameters, including the FAZ size, FAZ circularity, and superficial/deep/total retinal perfusion and vessel densities.

The significance level was set at p<0.05. All statistical analyses were performed using GraphPad Prism ver. 8.03 for Windows (GraphPad Software, San Diego, CA, USA). We included all possible outliers for statistical analysis as they may have clinical significance.

The study was conducted in accordance with the tenets of the Declaration of Helsinki, and the Institutional Review Board of Shinshu University approved the study (Ethics Review Approval Number: 4922). Informed consent was obtained using the opt-out method.

## Supporting information

S1 File (DOCX)
