# Supplementary material for: Application of optical coherence tomography angiography to assess systemic severity in patients with hereditary transthyretin amyloidosis
Source: PLoS One. 2022 Sep 26;17(9):e0275180. doi: 10.1371/journal.pone.0275180 (PMC9512205; doi:10.1371/journal.pone.0275180)
Supplement: S1 File — (DOCX) [file pone.0275180.s001.docx]

**Supporting information**

To quantify CC FD% with global mean binarization thresholding, CC slab images were imported into Fuji software, and then

select File>New>script, and paste the following;

run("8-bit");

setAutoThreshold("Mean");

//run("Threshold...");

run("Analyze Particles...", "summarize");

followed by select Language>IJ1 Macro and click "run.”

The mean choriocapillaris flow deficit percentage (CC FD%) with global mean binarization thresholding was 50.56±1.92, which was not significantly correlated with the systemic severity score (**S1 Figure.** Spearman's rank correlation: r=-4.9×10^-2^, p=0.778). The results of the subsequent multivariate analysis are shown in **S1 Table**.

**S1 Figure.** Spearman's rank correlation analysis showed no significant correlation between choriocapillaris flow deficit percentage (CC FD%) quantified with global mean binarization thresholding and the systemic severity score.

**S1 Table.** Univariate and multivariate analyses for the relationship between systemic severity scores and parameters including choriocapillaris flow deficit percentage calculated with global mean binarization thresholding

| **Parameters** | **Univariate Analysis** | | **Multivariate Analysis** | | |
| --- | --- | --- | --- | --- | --- |
|  | **R (95% CI)** | **P value** | **β (95% CI)** | **Test Statistic** | **P Value** |
| CC FD% (Global Mean) | -0.049 (-0.38 to 0.29) | 0.778 | -0.031 (-0.563 to 0.503) | 0.1164 | 0.908 |
| Age | -0.038 (-0.37 to 0.30) | 0.826 | -0.0079 (-0.093 to 0.077) | 0.1889 | 0.8513 |
| Signal Strength | -0.086 (-0.41 to 0.26) | 0.618 | -0.285 (-1.48 to 0.91) | 0.4849 | 0.6311 |

CC FD%, choriocapillaris flow deficit percentage; CI, confidence interval
